# Are Dental Caries Associated with Oxidative Stress in Saliva in Children and Adolescents? A Systematic Review

**DOI:** 10.3390/metabo12090858

**Published:** 2022-09-13

**Authors:** Yago Gecy de Sousa Né, Deborah Ribeiro Frazão, Leonardo Oliveira Bittencourt, Nathalia Carolina Fernandes Fagundes, Guido Marañón-Vásquez, Maria Elena Crespo-Lopez, Lucianne Cople Maia, Rafael Rodrigues Lima

**Affiliations:** 1 Laboratory of Functional and Structural Biology, Institute of Biological Sciences, Universidade Federal do Pará, Belem 66075-110, PA, Brazil; 2 School of Dentistry, Faculty of Medicine and Dentistry, University of Alberta, Edmonton, AB T6G 2R3, Canada or; 3 Department of Pediatric Dentistry and Orthodontics, School of Dentistry, Universidade Federal do Rio de Janeiro, Rio de Janeiro 21941-901, RJ, Brazil; 4 Laboratory of Molecular Pharmacology, Institute of Biological Sciences, Universidade Federal do Pará, Belem 66075-110, PA, Brazil

**Keywords:** dental caries, saliva, oxidative stress, systematic review

## Abstract

This systematic review aimed to assess whether dental caries is associated with oxidative salivary stress. The searches were carried out in electronic databases, including PubMed, Scopus, Web of Science, the Cochrane Library, LILACS, OpenGrey, and Google Scholar, without restrictions on the date of publication and language. The acronym PECO was used, in which the participants (P) were children and adolescents exposed (E) to dental caries compared (C) to those without dental caries, with the outcome (O) of modulation of oxidative biochemical parameters. After the search retrieval, the duplicates were removed, and the articles were evaluated by title and abstract, following the inclusion and exclusion criteria. Then, the papers were read and thoroughly assessed. After selection, the risk of bias assessment and qualitative synthesis were performed using the Newcastle-Ottawa Scale (NOS) for observational studies. The Grading of Recommendations, Assessment, Development, and Evaluation (GRADE) tool was used to assess the level of evidence. A total of 5790 studies were found, and 30 articles were considered eligible and were included for the qualitative synthesis and the level of evidence assessment. The studies showed an imbalance of the antioxidant and pro-oxidant parameters in individuals with dental caries, with primarily increases in both total antioxidant capacity and lipid peroxidation. Most articles showed a low risk of bias, having comparability as the main issue. When exploring through GRADE, a very low level of evidence was found. It was possible to observe an association between oxidative stress and dental caries, showing a disbalance of antioxidants and pro-oxidants, but the evidence level was still very low.

## 1. Introduction

Among oral diseases, the most prevalent chronic disease is dental caries [1]. Caries is a multifactorial disease that affects the hard tissues of teeth through metabolites produced by the microorganisms in the oral flora as a result of the imbalance between the demineralization and remineralization processes. Due to the high production of acids by aciduric/acidogenic bacteria, demineralization is more prevalent through frequent exposure to sugars in the biofilm [2].

Saliva, when constantly bathing the teeth and oral mucosa, works as a cleaning solution, having lubricating and buffering actions as well as acting as a reservoir of calcium and phosphate. These minerals are essential ions for the remineralization of the initial carious lesions through the process of remineralization and demineralization of dental enamel that occurs in the oral cavity (DES-RE process). The biochemical composition of saliva, which is supersaturated with the existing hydroxyapatite in tooth enamel, facilitates this mechanism by minimizing demineralization and favoring remineralization. In addition, saliva dilutes and neutralizes dietary acids and the bacterial metabolism of the biofilm [3,4]. Saliva has a significant role in the defense of the oral cavity as it has antimicrobial factors and antioxidant properties, acting like this in the control of dental caries [5,6,7].

The antioxidant properties of saliva provide a balance between free radicals, thus playing an essential role in protecting the oral cavity. Free radicals are highly unstable molecules, able to gain or lose electrons from other molecules to become more stable. They become unstable when they acquire electrons from nucleic acids, lipids, and proteins, causing a cascade of reactions that result in cellular damage. There are two main types of free radicals: reactive oxygen species (ROS) and reactive nitrogen species (NOS) [8]. The increase of ROS causes an imbalance, thus leading to oxidative stress and damage of macromolecules, such as proteins, nucleic acids, and lipids, which can cause necrosis and cell death [9,10,11].

Under normal circumstances, our bodies have a defense system that controls the amount of free radicals called antioxidants, which reduces the proliferation of ROS and ensures cell repair. The antioxidant system can be divided into enzymatic systems (such as superoxide dismutase and catalase), non-enzymatic systems (such as reduced glutathione, melatonin, and vitamin E), and systems of incorporation of free radicals into nitrogenous bases, through hydroxylated DNA bases and DNA repair enzymes [11]. The imbalance between these lines of defense and the production of free radicals leads to an excess of free radicals and damage to macromolecules (i.e., lipids, proteins, and DNA), a phenomenon known as oxidative stress [11].

Some biomarkers of oxidative damage have been found in individuals with dental caries, with modulations of antioxidant defense parameters, as well as pro-oxidants in saliva [7,10]. In addition, some studies have shown that children with dental caries have higher levels of protein in their saliva than children without dental caries. Oxidative stress biomarkers can be detected in saliva concentrations, so the levels of these biomarkers in saliva reflect specific oxidative pathways associated with dental caries [12]. Most studies on oxidative stress and saliva investigate parameters such as TAC, LPO, SOD, and GSH [13,14,15]. But from the numerous possibilities of parameters to be investigated, this systematic review seeks precisely to map the most diverse parameters already investigated, the methodological quality of the studies and the certainty of evidence presented with saliva studies.

## 2. Materials and Methods

### 2.1. Registration

This study was recorded in the Open Science Framework database under registration DOI 10.17605/OSF.IO/EB5S3 and performed according to the Preferential Reporting Requirements for Systematic Review (PRISMA) statement [16].

### 2.2. Eligibility Criteria

This systematic review was carried out with the PECO question strategy, covering observational studies. The participants (P) were children or adolescents aged 0 to 19 years with dental caries (E) compared to those without dental caries (C), and the outcome (O) modulation of oxidative biochemical parameters.

Inclusion criteria were observational studies that evaluated healthy children and adolescents who had at least one dental caries tooth and had performed some salivary oxidative biochemical analysis to verify whether or not there was an association between dental caries and modulation of oxidative biochemical parameters. Technical papers, clinical cases, literature reviews, guides, letters to the editor, opinion articles, and animal studies were excluded.

### 2.3. Information Source

The searches were conducted in the following electronic databases: PubMed, Scopus, the Cochrane Library, Web of Science, and LILACS. Regarding the grey literature, OpenGrey and Google Scholar were also assessed, without the restriction of language or year, until June 2022. A search strategy was pre-defined from a combination of MeSH and free terms related to children, adolescents, dental caries, oxidative stress, and saliva (Table S1). The previously defined terms were adapted to the syntax rules of each bibliographic database. An alert in each database was activated to identify articles that met our eligibility criteria and were published after the systematic searching in June 2022.

### 2.4. Search Strategy/Selection Process

The citations recorded were exported to a bibliographic reference manager (EndNote, version X9, Thomson Reuters, Philadelphia, PA, USA), in which duplicates were removed, automatically and manually. First, the articles were assessed by title and abstract. Then, the full text of the remaining items was reviewed to include all the articles that met our eligibility criteria. It also analyzed the references from the included studies to seek articles that met the inclusion criteria for being part of this systematic review.

All evaluations, including searches, study selection, and subsequent risk of bias assessment and data extraction, were performed independently by two reviewers (YGSN and DRF) and checked by a third-party disagreement evaluator (RRL).

In the case of missing data, contact was made with the authors, who were sent one e-mail per week for five consecutive weeks.

### 2.5. Data Items

After the study selection, the data was tabulated, extracting the following information: authors, country, year, study design, participants, population, number of participants, age, caries diagnosis, biochemical analysis method, biochemical parameters, and results.

The meta-analysis was not possible due to the great heterogeneity of the included studies, which used very different analyses to evaluate the oxidative stress parameters, some using protein corrections. They also used different wavelengths for spectrophotometer analysis, which makes it impossible to analyze the results together.

### 2.6. Study Risk of Bias Assessment

The evaluation of the quality and risk of bias of included studies was conducted using the Newcastle-Ottawa Scale (NOS) [17] for observational studies.

The NOS [17] comprises a checklist with three major bias domains: selection, comparability, and exposure. In the first section, the study is evaluated regarding the case definition, the representativeness of the cases, and the selection and definition of the controls. The second domain evaluates the comparability of cases and controls based on the design or analysis. The exposure section analyzes the ascertainment of exposure, the non-response rate, and whether the study used the same method of ascertainment for cases and controls. Therefore, reviews can get a maximum of nine stars—four stars for selection, two stars for comparability, and three stars for the outcome.

### 2.7. Certainty Assessment

The certainty of the evidence was assessed for the narratively synthesized results on modulation of oxidative biochemical parameters (TAC, LPO, and nitric oxide levels), using the Grading of Recommendations, Assessment, Development, and Evaluation Pro software (GRADEpro Guideline Development Tool, McMaster University and Evidence Prime, Hamilton, ON, Canada, 2021. Available online at gradepro.org (accessed on 11 June 2022)) [18]. The risk of bias, inconsistency, indirectness, imprecision, and the suspicion of publication bias were the contemplated aspects to rate the overall certainty of evidence [19,20].

## 3. Results

### 3.1. Selection and Characteristics of the Studies

A total of 5790 records were identified from the searches of the databases, and 2654 duplicates were found and removed. The remaining 3136 records were evaluated by title and abstract according to the eligibility criteria, and as a result, 3096 studies were excluded at this stage.

**Figure 1:**
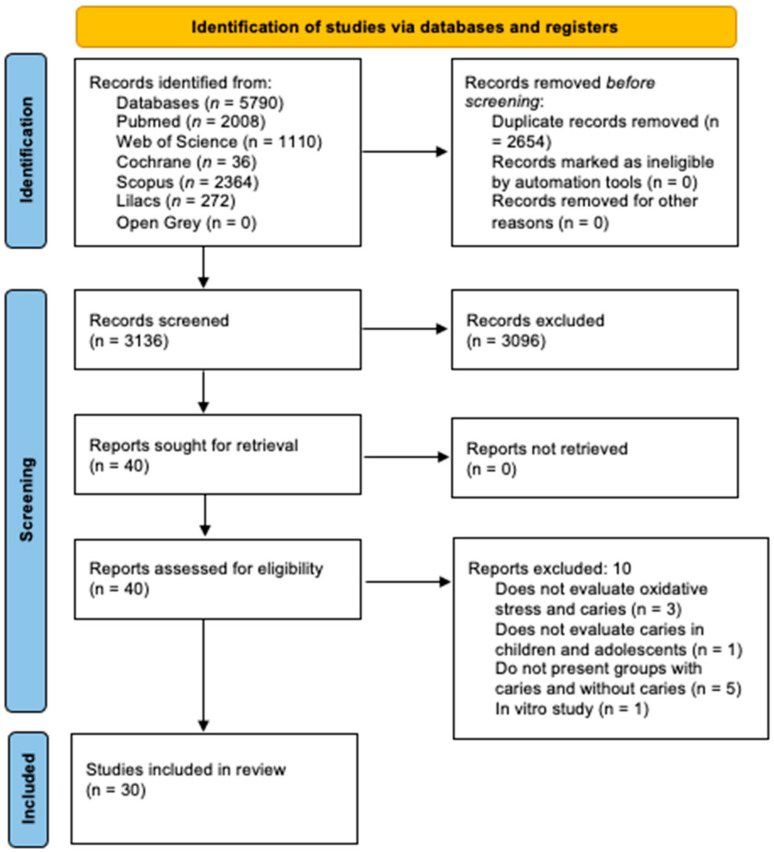
PRISMA 2020 flow diagram.

The remaining studies (*n* = 40) were assessed by reading the full text, and ten studies were excluded due to the following causes: three didn’t evaluate oxidative stress and caries, one didn’t evaluate caries in children and adolescents, five didn’t have caries free and caries active groups, and one is an in vitro study, conflicting with the previously established eligibility criteria. Finally, 30 studies were selected in this systematic review according to the eligibility criteria [10,13,14,15,21,22,23,24,25,26,27,28,29,30,31,32,33,34,35,36,37,38,39,40,41,42,43,44,45,46,47]. Results are presented in Figure 1.

### 3.2. Individual Results of Included Studies

Eleven of the included studies were case-control, and nineteen were cross-sectional studies. Nine of them were conducted in Iran [13,21,31,36,39,40,42,45,46], one in Saudi Arabia [22], twelve in India [23,26,27,30,32,33,34,35,37,43,44,47], one in Argentina [24], three in Brazil [15,25,41], three in Poland [14,28,30], and one in Turkey [38]. These studies evaluated the association of dental caries and oxidative stress by analyzing the following antioxidant parameters: total antioxidant capacity (TAC), reduced glutathione (GSH), glutathione peroxidase enzymes (GSH-px), ascorbic acid (vitamin C), superoxide dismutase (SOD), uric acid (UA), and catalase (CAT). As for the pro-oxidant parameters, they evaluated lipid peroxidation (LPO) and total nitrates/nitrites.

Among the 30 studies, 21 [13,14,15,21,22,23,24,25,26,28,29,30,31,32,33,34,35,36,38,45,46] evaluated the total antioxidant capacity (TAC). Some evaluated LPO, GSH, GSH-px, xanthine oxidase, vitamin C, CAT, SOD, oxidized glutathione (GSSG), and UA, and four evaluated the levels of nitric oxide and total nitrates/nitrites. All studies had children or adolescent patients with dental caries as a study group; also, saliva was collected to analyze oxidative stress in all studies.

Among the 21 [13,14,15,21,22,23,24,25,26,28,29,30,31,32,33,34,35,36,38,45,46] that evaluated the TAC, 16 [13,14,22,23,25,26,27,29,30,31,32,33,34,35,38,46] showed an increase in the group that had dental caries, four studies [24,28,36,45] showed that the control group had a higher TAC value in their saliva, and only one study [13] showed that there were no significant differences between the groups.

Only eight studies [13,24,25,35,39,40,44,45] evaluated LPO; six [13,25,35,39,40,44] found an increase in the exposed group; one [24] found that the LPO level was low in all groups; and one found no difference between groups [45]. In 1996, Corvalán et al., showed that the levels of xanthine oxidase, GSH-px, GSH, vitamin C, CAT, and SOD were higher in the control group. On the other hand, Silva et al., 2016 and Jurczak et al., 2017 showed that levels of SOD, UA, GSH, and GSSG were higher in the exposed group.

Due to the significant methodological heterogeneity of the studies, mainly concerning the method of analysis of the biochemical parameters observed in Table 1 in the analysis tab, it was impossible to perform the quantitative research through a meta-analysis.

### 3.3. Qualitative Assessment of Studies and Risk of Bias

In the risk of bias analysis using the NOS [17], among the 30 studies, 12 were case-control and 18 were cross-sectional. Among the case control studies [13,21,22,28,29,36,37,39,40,42,45] obtained stars in almost all domains (case definition adequate, representativeness of the cases, selection of controls, definition of controls, comparability, ascertainment of exposure, same method of ascertainment for cases and controls), just one domain (non-response rate) had problems in four studies, which makes these studies present a better methodological consistency (Table 2). The 19 cross-sectional studies [23,24,25,26,27,28,29,30,31,32,33,34,35,38,41,43,44,46,47] all obtained stars in almost all domains. Corvalán et al., 1996 [24] had problems in all domains except in selection. Selection has failed in more than one domain, which causes the risk of bias to become high (Table 3).

### 3.4. Certainty of Evidence

The narrative syntheses for the modulation of oxidative biochemical parameters showed a very low certainty of evidence. The synthesized results on the TAC were affected mainly by the inconsistency of the effect sizes of the studies assessed and the absence of overlap among their confidence intervals. The products on the MDA and NO levels were also affected by the inconsistency of the effect sizes and by the impression due to the limited number of participants evaluated (GRADE recommended rule of thumb threshold: sample sizes larger than 400). This last criterion used to downgrade the evidence’s certainty was also applied to all other outcomes, including a single study (GSH, GSSG, GSH/GSSG, SOD, UA).

It is essential to mention that, although it was considered that the syntheses were not affected by indirectness, these results do not provide direct evidence for a specific age group since the included studies evaluated individuals of different ages. Publication bias was unsuspected for all the outcomes. Table 4 shows the certainty assessment of the most relevant results.

## 4. Discussion

In this systematic review, 30 articles were found. All of them showed an imbalance in pro-oxidants and antioxidants in children or adolescents with caries, suggesting an association of caries with salivary oxidative stress. The main parameter evaluated was the TAC, which was increased in the group with caries in 16 of the selected articles. Regarding the methodological quality of the studies, 29 of them scored in all domains; however, the level of evidence was very low, indicating that the association of oxidative stress with caries cannot be determined with certainty.

Saliva proved to be a fluid with a high capacity to detect molecules that can act as biomarkers of several oral diseases, such as periodontitis and dental caries [7]. It also serves as a potent means of diagnosing oxidative stress in saliva since we find markers of oxidative stress in saliva, which causes saliva to show a reflection of the changes that occur both in the oral cavity and in the entire organism. It is also related to the balance of both pH and antioxidants in the oral cavity [7]. In this systematic review, these articles investigate the parameters associated with the modulation of antioxidant defenses (TAC, GSH, vitamin C, SOD, UA, and CAT) and pro-oxidants (LPO and NO) in the saliva of patients with dental caries. Of these parameters, those that were shown to be altered in these articles were mainly TAC, LPO, SOD, GSH, and UA, which were increased in the groups with active decay, and the levels of nitrates and nitrites were lower in the group with dental decay. These changes show us that dental caries are associated with antioxidant defense responses and an imbalance in the oxidative process.

Among the pro-oxidant factors evaluated, the chosen studies evaluated LPO and nitrates/nitrites. Some studies included in this review showed that there was an increase in lipid peroxidation (LPO) when they analyzed malondialdehyde (MDA) [13,25,35]. MDA is the final product of LPO and is related to salivary oxidative stress, thus showing whether there is an imbalance of the pro-oxidant and antioxidant systems [48].

Interestingly, no evaluation has been carried out to analyze the oxidative damage of proteins and DNA, although the latter can lead to even more deleterious consequences than damage to lipids. The sulfur-containing amino acids cysteine and methionine are particularly susceptible to ROS, and the oxidative damage to proteins can be measured by protein carbonylation [49]. DNA damage by oxidative stress includes base modifications, basic sites, and strand breaks. While guanine usually pairs with cytosine, oxidized guanosines (8-hydroxy-2’-deoxyguanosine -8-OHdG-, and 8-oxo-7,8-dihydro-2’-deoxyguanosine -8-oxodG-), which are the most frequent type of oxidative base damage, may cause mispairing with adenine through a conformational change. This is a classical route to induced mutations that is also used to evaluate oxidative DNA damage by quantitation of 8-OHdG and 8-oxodG as resulting byproducts [50]. It is somewhat surprising that no study has evaluated the possible oxidative genotoxicity of caries, considering that salivary DNA damage has been used as a marker in other studies [51,52,53]. Furthermore, considering the vulnerable population being analyzed (children and adolescents), it is urgent to obtain reliable results about the possible genotoxic consequences of caries, because this population usually has a long lifespan and, consequently, high probability of accumulating mutations that eventually lead to carcinogenesis.

Nitric oxide is a free radical and is one of the smallest and simplest biosynthesized molecules [54]. NO is synthesized through the oxidation of one of the two guanine nitrogen bases of L-arginine (an essential amino acid for many functions in our body), which is converted into L-citrulline. This reaction is catalyzed by the enzyme NO-synthase (NOS) [55,56]. There are several isoforms of NO-synthase. In the oral cavity, the inducible NOS (i-NOS) that performs NO synthesis in the oral cavity is produced by macrophages and other cells activated by cytokines, and this enzyme is expressed in the salivary glands [56,57]. The increase in NO is related to individuals who have poor hygiene and dental caries [58]. Studies that analyzed NO showed that levels were low in groups with dental caries [27,37]. Nitric oxide participates in the defense of the oral cavity against bacterial multiplication. It is observed that the increase in NO levels in saliva can be a defense mechanism when there is neglect of oral hygiene and an increase in dental caries in individuals [15].

Among the antioxidant factors evaluated, the chosen studies evaluated TAC, GSH, vitamin C, SOD, UA, and CAT. The articles selected in this study that analyzed the TAC showed an increase in these antioxidants in individuals with dental caries [13,14,22,23,25,26,27,29,30,31,32,33,34,35,38,46]. TAC evaluation is one of the fastest, cheapest, and most accessible methods, thus facilitating the general observation of all antioxidants [59]. The TAC shows the combined effect of antioxidants, mainly non-enzymatic, present in the plasma and body fluids, such as saliva, since they all work together [59]. However, the analysis of TAC has some limitations, mainly because it provides limited information on specific mechanisms of free radical scavenging and therefore cannot provide the contribution of individual antioxidant species to the pathology of specific diseases [59]. In addition, the different systems used to measure TAC appear to be sensitive to different antioxidants, and the oxidative damage index used to define the free radical-induced oxidation process is also different. So, the data between experiments may not be comparable [59]. Thus, the increase in TAC shows an imbalance between antioxidants and pro-oxidants in the oral cavity.

Another analysis that was made in the studies was that of UA. The studies that performed this analysis observed an increase in this antioxidant [15,25]. UA is another non-enzymatic antioxidant. UA is very efficient in eliminating ROS in both the plasma and saliva, which contributes to minimizing the damage caused by the possible oxidative imbalance caused by dental caries [60].

To observe the enzymatic antioxidant system, we performed an analysis of superoxide dismutase (SOD) [61]. SOD catalyzes the dismutation of the superoxide anion into oxygen and hydrogen peroxide so that the superoxide anion causes a decrease in the bioavailability of nitric oxide (NO) [61,62,63]. The articles that analyzed SOD showed an increase in the group with dental caries, which are related to the stage at which dental caries are found. Higher levels of SOD are found in the most severe cases of caries, thereby illustrating an attempt to restore an oxidative balance [15].

Glutathione (GSH) is the most critical low molecular weight antioxidant synthesized in animal cells and is found mainly in the cytosol [64]. Due to the cysteine residues, GSH ends up being oxidized non-enzymatically to GSSG by free radicals, which causes a loss of GSH within the cells. So, GSH/GSSG is the leading redox pair that determines the antioxidant capacities of cells and fluid [65]. GSH is responsible for directly or indirectly neutralizing free radicals through the reaction catalyzed by GPx peroxidase and other peroxidases, thus neutralizing H2O2 and nitric oxide [65]. The levels of GSH are altered in the studies in which it was analyzed, also showing an increase in the group with caries, indicating the body’s need to try to fight and neutralize free radicals [14].

It is worth pointing out that the antioxidant capacity measured in the various studies elected may suffer modulation by supplementation with antioxidants and even by diet. Although the articles do not report this, nor was it the object of study in their investigations, these are points that deserve highlighting. Vijayavel et al., 2006 showed that, ascorbic acid and α-tocopherol supplementation reduced lipid peroxidation and increased enzymatic (Superoxide dismutase, Catalase, and Glutathione peroxidase) and nonenzymatic (Glutathione) antioxidants, indicating a possible activity against free radicals [66]. Antioxidants such as ascorbic acid and α-tocopherol can undergo oxidation and thereby provide electrons that will be used by the oxidized glutathione through the action of glutathione reductase. In the same way, fruits and vegetables provides supplementation of vitamins C and E, carotenoids, and flavonoids, which are nutrients with high antioxidant capacity and can act together with endogenous antioxidants against free radicals generated by physiological conditions or by exposure to free radical generating agents [67,68].

As shown by Araujo et al., 2020, antioxidants are altered depending on the stage of caries. Early diagnosis of dental caries is crucial, making the treatment less invasive and more productive [69]. Thus, based on the International Caries Detection and Assessment System (ICDAS) and the International Caries Classification and Management System (ICCMS ™), which were the methods found in the included articles, it allows dentists to have a guide to measure the risk of caries accordingly. Clinical practice is more effective when information is shared with other professionals [48]. The level of caries aggression is directly linked to the imbalance of the pro-oxidant and antioxidant systems; children with severe caries had a higher level of TAC, SOD, UA, MDA, and GSH and lower levels of NO, thus showing a direct relationship between the severity of caries and the activity index of the antioxidant system [13,15,26,37]. There is then an increase in total antioxidant levels to minimize the oxidative damage caused [15].

The levels of antioxidants and pro-oxidants are also altered depending on the age of the individual, which is also related to the severity of this pathology. The study by Araujo et al., 2020 showed that TAC levels could change depending on the individual’s age; children have a greater TAC when compared to adolescents. Children with caries in the early stages have more cariogenic bacteria, such as Streptococcus mutans, which have high acidogenic activity and are not found in adolescents with caries [45].

The degree of association of the biochemical oxidative parameters is altered according to age [46]. Salman et al., 2021 conducted the analysis by observing groups of children and adolescents. When they analyzed children from 3 to 12 years, there was no statistical difference that showed an association between the markers of oxidative stress and dental caries. This may be because the immune system is not yet fully formed, which makes the inflammatory process incomplete. When observed in adolescents aged 13 to 18 years, there was a significant decrease in the total antioxidant capacity in the group with caries, which may possibly be associated with the excess of free radicals [46].

The articles included in this systematic review obtained scores in almost all the domains evaluated but presented comparability problems. Age directly influences the antioxidant capacity of saliva; another factor that is directly related to antioxidant capacity is eating habits, in which a healthy diet composed of probiotics and antioxidants derived from fruits and vegetables increases these defenses against oxidative stress. Bad habits, such as smoking, drinking, and eating fast food, are related to a decrease in these defenses, in which it was observed that adolescents had worse habits [70].

The level of evidence of studies carried out jointly by GRADE was considered very low. This tool assesses whether the evidence from the study selection is strong enough to conclude the association of oxidative stress with dental caries. GRADE parameters consider less than 400 participants as a qualifier for severe imprecision. The variation in effect sizes also contributed to the fact that the level of evidence was very low. Larger sample sizes and a combination of more sensitive biochemical analyses are essential to properly observe whether there is a direct relationship between caries and oxidative stress.

When all studies were analyzed, it was possible to observe that there was an agreement between the studies, showing that there is an association between oxidative stress and caries activity in children and adolescents, with an increase in the biochemical parameters evaluated in individuals with caries being also observed, especially those who had caries in a more advanced stage.

However, this systematic review showed that the biochemical modulations between pro-oxidants and antioxidants that occur in saliva are associated with the presence of dental caries. Despite this association, most of the studies do not subdivide into ages, which may affect the results obtained, due to the relationship between age and salivary pro-oxidant and antioxidant levels. Another limitation of this review is that many of the studies included, only evaluated the total antioxidant capacity, which does not give us a complete picture of the biochemical modulations of saliva.

Despite the limitations, this systematic review has shown that with possible advances in the analysis of oxidative biochemical parameters in saliva, we can suggest possible carious formation at an early stage and thus prevent the disease from progressing and showed the biochemical modulation of saliva against carious disease at different ages. More studies with more parameters such as GSH, SOD, LPO, UA, and NO together to give us more details about the relationship between dental caries and oxidative stress in saliva are needed.

## 5. Conclusions

It was possible to observe a biochemical modulation linked to caries and to the pro-oxidant and antioxidant systems. The articles showed a high level of antioxidant response by increasing mainly TAC in the caries group, but we also saw an increase in lipid damage by the LPO parameter in the same group, showing a disbalance of antioxidants and pro-oxidants. We could see a very low level of evidence. Future studies with more combined analyses will give a clearer picture of the association of caries with oxidative stress.

## Figures and Tables

**Table 1:** Extraction table.

Author/Country/Year	Study Design	N	Age (Years)	Caries Diagnostic	Biochemical Analysis Method	Biochemical Method	Results
Ahmadi-Motamayel et al./Iran/2013 [21]	Case-control	100 50—Caries free 50—Caries active	15–17	DMFT	TAC	Antioxidant commercial assay kit (Cayman Chemical, ABTS oxidation inhibition, wavelength: 405 nm).	The exposed group (active caries) showed higher TAC levels in comparison to the control group (caries-free; *p* < 0.001). Regarding the inter- and intragender comparisons, there was no difference between females in the exposed and control groups. The male participants showed higher TAC levels in the active caries group when compared to those in the caries-free group.
Ahmadi-Motamayel et al./Iran/2018 [13]	Case-control	118 56—Caries free 62—Caries active	15–19	DMFT	TAC and LPO	TAC: Ferric Ion Reducing Antioxidant Power Assay (wavelength 593 nm) LPO: Thiobarbituric Acid Reactive Species assay (wavelength: 520 nm).	There was no statistical difference between the exposed and control groups regarding the TAC levels. The LPO levels were higher in the exposed group (active caries) when compared to those in the control group (caries-free; *p* = 0.001). Considering the comparison between genders, the LPO levels were higher in male participants (*p* = 0.02) when compared to female participants.
Alanazi et al./Saudi Arabia/2018 [22]	Case-control	40 20—Caries free 20—Caries active	5.13 ± 0.79	Not informed	TAC	Evaluated by a commercial kit of Oxygen Radical Absorbance Antioxidant Assay (Zen-Bio ORAC™, AMS Biotechnology).	The TAC levels were higher in the exposed group (severe early childhood caries) when compared to those in the control group (*p* = 0.003).
Aliakbarpour et al./Iran/2021 [39]	Case-control	90 30—Caries free 60—Caries active	3–5	DMFS	LPO	Evaluataed by the level of TBARS in saliva.	The mean of salivary TBARS in saliva was higher in the caries active group compared to the caries free group (*p* < 0.001).
Amrollahi et al./Iran/2021 [40]	Case-Control	84 42—Caries free 42—Caries active	4–6	DMFT	LPO	The salivary MDA was analyzed using a commercial kit (ZellBio GmbH, Germany), according to the manufacturer’s instructions. The absorbance was obtained with a microplate reader/ELISA reader at 535 nm.	The mean salivary MDA in the ECC group (4.8 ± 0.6) was significantly higher than that in the caries-free group (2.9 ± 0.5) (*p* = 0.01).
Araujo et al./Brazil/2020 [15]	Cross-sectional	120 30—Caries free 90—Caries active	1–3	ICCMS™ index	Total Proteins, LPO, TAC, SOD, and uric acid.	Total Proteins: Biuret method (wavelength 660 nm); LPO: Thiobarbituric Acid Reactive Species assay (wavelength 535 nm); TAC: Ferric Ion Reducing Antioxidant Power assay (wavelength 595 nm); SOD: Pyrogallol autoxidation reducing capacity (wavelength 420 nm); Uric acid: Commercial kit assay (Labtest Diagnóstica).	Total protein levels were higher in the extensive caries groups when compared to those in the other groups (*p* < 0.001). Moreover, there was a moderate positive correlation between protein levels and caries severity (Spearman’s r = 0: 7084, *p* < 0.0001); The LPO levels were lower in the extensive caries group when compared to those in the other groups (*p* < 0.0001). Moreover, there was a strong negative correlation between LPO levels and caries severity (Spearman’s r = −0.8570, *p* < 0.0001). The TAC levels were higher in the extensive caries group when compared to those in the other groups (*p* < 0.001), and there was a strong positive correlation between caries severity and TAC levels (Spearman’s r = 0.8.425, *p* < 0.0001); The SOD activity was higher in the extensive caries group when compared to those in the other groups (*p* < 0.001), and there was a strong and positive correlation between caries severity and SOD activity (Spearman’s r = 0.7320, *p* < 0.0001); The salivary uric acid levels were higher in the extensive caries group compared to those in the other groups (*p* < 0.0001). Also, there was a weak and positive correlation between uric acid levels (corrected by protein levels) and caries severity (Spearman’s r = 0.4659, *p* < 0.0001).
Banda et al./India/2016 [23]	Cross-sectional	60 30—Caries free 30—Caries active	6–12	DMFT	TAC	Phosphomolybdenum assay (wavelength 695 nm).	There was a strong and positive correlation between the DMFT score and TAC level.
Corvalán et al./Argentina/1996 [24]	Cross-sectional	95 13—Caries free 82—Caries active	6–14	Not informed	Xanthine oxidase, ROS content, LPO, GSH-px, GSH, vitamin C, CAT, SOD, and TAC	The parameters were assessed by spectrophotometric methods.	The xanthine oxidase and ROS levels were absent in the caries-free group but present in the active caries group. The TAC and SOD levels were higher in the caries-free group and reduced in the active caries group. The LPO levels, assessed by malondialdehyde levels, were low in all groups. Vitamin C and GSH were present in the caries-free group, and their values were recorded in the caries groups. The GSH-px levels were lower in patients with active caries. CAT activity was absent in all groups.
Eagappan et al./India/2016 [37]	Case-control	120 40—Caries free 80—Caries active	4–5	DMFS	Total nitrites and nitrates	Griess reaction method (wavelength 540 nm).	The mean concentration of nitrites and nitrates was lower in the exposed group in both conditions: early childhood caries and severe early childhood caries.
Farghaly et al./Brasil/2013 [41]	Cross-sectional	46 28—Caries free 18—Caries active	4–6	DMFS	Total salivary peroxidase activity	The peroxidase activity was evaluated by the variation of absorbance measured in a Beckman DU-68 spectrophotometer at 460 nm.	There was no statistical difference between the caries free group and the caries active group (*p* = 0.425), concerning the total salivary peroxidase activity.
Hegde et al./India/2008 [27]	Cross-sectional	120 60—Caries free 60—Caries active	6–12	DMFT	Total nitrites and nitrates	Griess reaction method.	The exposed group (active caries) had lower levels of total nitrites and nitrates when compared to those in the control group. There was a positive correlation between nitrites/nitrates concentration and age.
Hegde et al./India/2009 [26]	Cross-sectional	100 50—Caries free 50—Caries active	6–12	The dental caries status was assessed using the WHO Oral Assessment Form.	TAC	Assessed by thiobarbituric reactive species production inhibition.	In both situations, the exposed groups, early childhood caries and rampant caries, had higher TAC levels than in the control groups (*p* < 0.05).
Hendi et al./Iran/2019 [42]	Case-Control	100 50—Caries free 50—Caries active	15–17	A senior dental student performed all the intraoral examinations. Dental mirrors and explorers were used for the detection of caries.	SOD, UA, GSH-Px, CAT, Peroxidase	SOD: RANSOD kit (Randox Laboratories Ltd., Crumlin UK) UA: Pars Azmun Co. kit (Tehran, Iran) with spectrophotometry GSH-Px: RANSEL kit (Randox Laboratories Ltd., Crumlin, UK) CAT: Spectrophotometer.	The results showed higher UA(*p* = 0.641), CAT (*p* = 0.491), and GSHPx (*p* = 0.004), Prox (*p* = 0.072), and lower SOD (*p* = 0.935) in the caries active group compared to the caries-free group. However, only GSHPx increased in the caries-active subjects was significant.
Jurczak et al./Poland/2017 [14]	Cross-sectional	81 27—Caries free 54—Caries active	2–5	ICDAS	GSH, GSSG, and TAC	TAC: Ferric Ion Reduction Power, wavelength 593 nm; GSH and GSSG: Reaction of Griffith’s method with Tietze’s modification, wavelength 412 nm.	All the biochemical parameters were higher in the active caries group (*p* < 0.001).
Karthika et al./India/2021 [43]	Cross-sectional	100 50—Caries free 50—Caries active	6–12	DMFT	Vitamin E, GPx	Vitamin E: spectrophotometer GPx: spectrophotometer.	There was a decrease in vitamin E levels in the caries active (1.25 ± 0.01) group when compared with the caries-free (1.37 ± 0.01) group. The mean GPx levels decreased in the values in caries active (0.53 ± 0.08) group when compared to the caries-free (1.62 ± 0.14) group.
Krawczyk et al./Poland/2014 [28]	Case-control	113 25—Caries free 88—Caries active	15–17	DMFT, oral hygiene index	TAC	Antioxidant commercial assay kit (Randox Laboratories Ltd. ABTS oxidation inhibition method, wavelength 600 nm).	The salivary antioxidant status in the exposed group was lower than the levels found in the control group.
Krawczyk et al./Poland/2012 [29]	Case-control	60 30—Caries free 30—Caries active	16–18	DMFT, DMFS	TAC	Antioxidant commercial assay kit (Randox Laboratories Ltd. ABTS oxidation inhibition method, wavelength 600 nm).	The salivary TAC levels in the exposed group (active caries) were higher than those in the control group (caries-free). Moreover, the TAC level was higher in younger participants.
Kumar et al./India/2011 [30]	Cross-sectional	100 50—Caries free 50—Caries active	3–5	DMFT	TAC	Antioxidant commercial assay kit (Cayman Chemical, ABTS oxidation inhibition, wavelength 405 nm or 750 nm).	The salivary TAC levels in the exposed group (active caries) were higher than those in the control group (caries-free).
Mahjoub et al./Iran/2014 [31]	Cross-sectional	80 40—Caries free 40—Caries active	3–5	DMFS	TAC and Total Protein Levels	TAC: Ferric Ion Reducing Antioxidant Power assay (wavelength 593 nm); Total Protein Levels: Bradford’s method (wavelength 595 nm).	The salivary TAC levels were higher in the exposed group (active caries) when compared to those in the control group (caries-free; *p* = 0.025). Moreover, the salivary protein levels were higher in the exposed group (*p* = 0.033).
Muchandi et al./India/2015 [32]	Cross-sectional	50 25—Caries free 25—Caries active	3–5	Preliminary examination	TAC	They were assessed by thiobarbituric acid reactive species production inhibition (wavelength 532 nm).	The salivary TAC levels in the exposed group (severe early childhood caries) were higher than those in the control group (caries-free).
Pandey et al./India/2015 [33]	Cross-sectional	120 60—Caries free 60—Caries active	7–15	DMFS	TAC and Total Protein Levels	TAC: Ferric Ion Reducing Antioxidant Power assay (wavelength 600 nm) Total Protein Levels: Biuret method (wavelength 545 nm).	The salivary TAC levels in the exposed group (active caries) were higher than those in the control group (caries-free). Moreover, the total protein levels were higher than in the control group.
Prabhakar et al./India/2009 [34]	Cross-sectional	120 60—Caries free 60—Caries active	7–14	DMFS	TAC and Total Protein Levels	Human diagnostic kit (Germany).	The salivary TAC and total protein levels increased significantly in the exposed group when compared to those in the control group.
Pyati et al./India/2018 [35]	Cross-sectional	100 50—Caries free 50—Caries active	6–12	DMFS	TAC, LPO, and Total Protein Levels	TAC: Assessed by thiobarbituric reactive species production inhibition (wavelength 532 nm); LPO: Thiobarbituric acid reactive species (wavelength 530 nm); Total Protein Levels: Biuret method (wavelength 545 nm).	The TAC and MDA levels increased in children with active caries when compared to caries-free controls (*p* < 0.05). Moreover, the total protein levels also increased in the active caries group when compared to those in the control group (*p* = 0.017).
Rahmani et al./Iran/2015 [36]	Case-control	120 60—Caries free 60—Caries active	14–18	DMFT	TAC	Antioxidant commercial assay kit (ZellBio kit, Ferric Reduction Antioxidant Power method, wavelength 520 nm).	The TAC levels were significantly lower in patients with dental caries when compared to patients without caries.
Ravikumar et al./India/2021 [44]	Cross-Sectional	60 20—Caries free 40—Caries active	3–6	DMFS	LPO	LPO: Spectrophotometer.	The caries active groups showed higher LPO compared to the caries free groups (*p* < 0.05).
Salman et al./Iran/2021 [45]	Case-control	163 85—Caries free 78—Caries active	3–18	Caries Index	LPO, TAC	LPO: TBARS TAC: Spectrophotometer.	When generally observed, LPO and TAC levels did not present significant differences. When only adolescents from 13 to 18 years of age were observed, the TAC showed a significant decrease in the group with caries when compared with the group without caries of the same age.
Shaki et al./Iran//2020 [46]	Cross-sectional	80 40—Caries free 40—Caries active	3–5	DMFT	TAC, NO	TAC: Ferric reducing antioxidant power (FRAP) NO: Commercial kits based on the Griess reagent.	The TAC was significantly higher in the active caries group when compared to control group (*p* < 0.05). The NO level were lower in the group with caries active compares to control group (*p* < 0.001).
Da Silva et al./Brazil/2016 [25]	Cross-sectional	60 30—Caries free 30—Caries active	0–3	DMFS	LPO, TAC, SOD, and Uric acid	LPO: Thiobarbituric acid reactive substance (wavelength 535 nm); TAC: Ferric reducing antioxidant power assay (wavelength 595 nm); SOD: Pyrogallol autoxidation reducing capacity (wavelength 420 nm); Uric acid: Commercial kit assay (Labtest Diagnóstica).	The TAC was significantly higher in the active caries group when compared to that in the control group (*p* < 0.05). LPO, SOD, and uric acid levels were more elevated in the active caries group when compared to those in the control group (*p* < 0.05).
Syed et al./India/2016 [47]	Cross-sectional	100 50—Caries free 50—Caries active	6–12	DMFT	NO	NO: Griess reaction method.	The salivary NO level was significantly higher in the caries free group (581.3 ± 134.6) compared to the caries active group (335.4 ± 111.2) (*p* = 0.000).
Tulonuglo et al./Turkey/2006 [38]	Cross-sectional	80 40—Caries free 40—Caries active	7–15	DMFS	TAC and Total Protein Levels	TAC: ABTS oxidation inhibition method Total Protein Levels: Biuret method (wavelength 545 nm).	TAC and total protein levels were higher in the group with active caries, except for girls aged 11–15.

TP: Total protein; TAC: Total antioxidant capacity; SOD: Superoxide dismutase; UA: Uric Acid; LPO: Lipid peroxidation; ICDAS: International Caries Detection and Assessment System; GSH: Reduced glutathione; GSSG: Oxidized glutathione; GSH-Px: Glutathione peroxidase enzyme; ABTS: 2,2′-azino-di-(3-ethyl-benzothiazoline 6-sulphonate); DMFS: Decayed, missing, filled surfaces; DMFT; Vit C: Ascorbic acid; ROS: Reactive oxygen species, GPx: Glutathione Peroxidase.

**Table 2:** Newcastle-Ottawa tool for case-control studies.

Authors	Selection				Comparability	Exposure		
	Case Definition Adequate	Representativeness of the Cases	Selection of Controls	Definition of Controls	Control vs. Case	Ascertainment of Exposure	Same Method of Ascertainment for Cases and Controls	Non-Response Rate
Ahmadi et al., 2013 [21]	*	-	*	*	*	*	*	-
Ahmadi et al., 2018 [13]	*	*	*	*	**	*	*	-
Alanazi et al., 2018 [22]	-	*	-	*	*	*	*	-
Aliakbarpour et al., 2021 [39]	*	*	*	*	**	*	*	*
Amrollahi et al., 2021 [40]	*	*	*	*	**	*	*	*
Eagappan et al., 2016 [37]	*	*	*	*	*	*	*	*
Hendi et al., 2019 [42]	*	*	*	*	*	*	*	*
Krawczyk et al., 2014 [28]	-	*	*	*	*	*	*	*
Krawczyk et al., 2012 [29]	*	*	*	*	**	*	*	*
Kumar et al., 2011 [30]	*	*	*	*	**	*	*	*
Rahmani et al., 2015 [36]	*	*	*	*	**	*	*	-
Salman et al., 2021 [45]	*	*	*	*	**	*	*	*

The symbol * was adopted to refer to the number of points/stars attributed to each category.

**Table 3:** Newcastle-Ottawa for cross-sectional studies.

Authors	Selection				Comparability	Exposure	
	Case Definition Adequate	Sample Size	Non-Respondents	Ascertainment of the Exposure (Risk Factor)	Control vs. Case	Ascertainment of Outcome	Statistical Test
Araujo et al., 2020 [15]	*	*	*	*	**	**	*
Banda et al., 2016 [23]	*	*	*	*	**	**	*
Covalán et al., 1996 [24]	*	*	-	*	*	*	*
Farghaly et al., 2013 [41]	*	-	*	**	**	**	*
Hegde et al., 2008 [27]	*	*	-	*	**	**	*
Hegde et al., 2009 [26]	*	*	-	*	**	**	*
Jurczak et al., 2017 [14]	*	*	*	*	**	**	*
Karthika et al., 2021 [43]	*	*	*	**	**	**	*
Mahjoub et al., 2014 [31]	*	*	*	*	**	**	*
Muchandi et al., 2015 [32]	*	*	*	*	*	**	*
Pandey et al., 2015 [33]	*	-	*	*	**	**	*
Prabhakar et al., 2009 [34]	*	*	*	*	**	**	*
Pyati et al., 2018 [35]	*	*	*	**	**	**	*
Ravikumar et al., 2021 [44]	*	*	*	**	**	**	*
Shaki et al., 2020 [46]	*	*	*	**	**	**	*
Da Silva et al., 2016 [25]	*	*	*	*	**	**	*
Syed et al., 2016 [47]	*	*	*	**	**	**	*
Tulunoglui et al., 2006 [35]	*	*	*	*	**	**	*

The symbol * was adopted to refer to the number of points/stars attributed to each category.

**Table 4:** Assessment of the certainty of evidence.

Certainty Assessment							Effect	Certainty
N° of Datasets	Design of the Studies	Risk of Bias	Inconsistency	Indirectness	Imprecision	Other Considerations	(Summary Narrative Description)	
Total Antioxidant Capacity (TAC)								
19	Observational studies	Not serious	Serious ^a^	Not serious	Not serious	None	For most studies (14 of 19), the TAC was higher in individuals with dental caries than in those without caries.	⨁◯◯◯ VERY LOW
Lipid Peroxidation (LPO)								
7	Observational studies	Not serious	Serious ^a^	Not serious	Serious ^b^	None	Most studies (6 of 7) reported higher levels of LPO for individuals with dental caries.	⨁◯◯◯ VERY LOW
Nitrate/Nitrite (NO) Levels								
4	Observational studies	Not serious	Serious ^a^	Not serious	Serious ^b^	None	All studies showed higher nitrate/nitrite levels in the caries-free group.	⨁◯◯◯ VERY LOW

^a^. The certainty of the evidence is downgraded by one level due to the variation in the effect size. There is no overlap between the confidence intervals. ^b^. The certainty of the evidence is downgraded by one level because the total number of individuals included in the synthesis is limited (GRADE recommended rule of thumb threshold: sample sizes larger than 400). The score to GRADE certainty vary from High to very low, ranked as 1–4. ⨁ symbol represents a full score (equivalent to one) and ◯ represents a zero score.

## Data Availability

The data presented in this study are available in article and Supplementary Material.

## Supplementary Materials

The following supporting information can be downloaded at: https://www.mdpi.com/article/10.3390/metabo12090858/s1, Table S1: Search strategies.
