# Supplementary material for: Are Dental Caries Associated with Oxidative Stress in Saliva in Children and Adolescents? A Systematic Review
Source: Metabolites. 2022 Sep 13;12(9):858. doi: 10.3390/metabo12090858 (PMC9502212; doi:10.3390/metabo12090858)
Supplement: Supplementary file 1 [file metabolites-12-00858-s001.zip › metabolites-1818195-supplementary.pdf]

Supplementary Table S1: Search strategies

Database	Search Strategy
PubMed	#1- (((((((((((((Humans[MeSH Terms]) OR (Humans)) OR (Man, Modern)) OR (Modern Man)) OR (Man (Taxonomy)) OR (Homo sapiens)) OR (Human)) OR (Dentition permanent[MeSH Terms])) OR (Dentition permanent)) OR (Permanent Dentition)) OR (Dentition, Secondary)) OR (Secondary Dentition)) OR (Dentition, Adult)) OR (Adult Dentition)<br>#2- ((((((((((((((((((((((((Dental Enamel Hypoplasia[MeSH Terms]) OR (Dental Enamel Hypoplasia)) OR (Hypoplastic Enamel)) OR (Enamel, Hypoplastic)) OR (Enamel Hypoplasia, Dental)) OR (Hypoplasia, Dental Enamel)) OR (Enamel Agenesis)) OR (Ageneses, Enamel)) OR (Agenesis, Enamel)) OR (Enamel Ageneses)) OR (Enamel Hypoplasia)) OR (Enamel Hypoplasias)) OR (Hypoplasia, Enamel)) OR (Hypoplasias, Enamel)) OR (Molar Incisor Hypomineralization)) OR (Hypomineralization, Molar Incisor)) OR (Molar Incisor Hypomineralization)) OR (Enamel)) OR (Tooth Demineralization[MeSH Terms])) OR (Tooth Demineralization)) OR (Tooth Hypomineralization)) OR (Hypomineralization, Tooth)) OR (Hypomineralizations, Tooth)) OR (Tooth Hypomineralizations)) OR (Demineralization, Tooth)<br>#3- (((Respiratory Tract Diseases[MeSH Terms]) OR (Respiratory Tract Diseases)) OR (Disease, Respiratory Tract)) OR (Diseases, Respiratory Tract)) OR (Respiratory Tract Disease)) OR (Tract Disease, Respiratory)) OR (Tract Diseases, Respiratory)) OR (Rhinosinusitis)) OR (Acute respiratory infection)) OR (Respiratory disease)) OR (Bronchial Diseases[MeSH Terms])) OR (Bronchial Diseases)) OR (Bronchial Disease)) OR (Disease, Bronchial)) OR (Diseases, Bronchial)) OR (Laryngeal Diseases[MeSH Terms])) OR (Laryngeal Diseases)) OR (Disease, Laryngeal)) OR (Diseases, Laryngeal)) OR (Laryngeal Disease)) OR (Larynx Diseases)) OR (Disease, Larynx)) OR (Diseases, Larynx)) OR (Larynx Disease)) OR (Laryngeal Perichondritis)) OR (Laryngeal Perichondritides)) OR (Perichondritides, Laryngea)) OR (Perichondritis, Laryngeal)) OR (Lung Diseases[MeSH Terms])) OR (Lung Diseases)) OR (Disease, Lung)) OR (Diseases, Lung)) OR (Lung Disease)) OR (Pulmonary Disease)) OR (Disease, Pulmonary)) OR (Diseases, Pulmonary)) OR (Pulmonary Diseases)) OR (Respiratory Tract Infections[MeSH Terms])) OR (Respiratory Tract Infections)) OR (Infection, Respiratory Tract)) OR (Respiratory Tract Infection)) OR (Respiratory Infections)) OR (Infections, Respiratory)) OR (Infections, Respiratory Tract)) OR (Upper Respiratory Tract Infections)) OR (Infections, Upper Respiratory Tract)) OR (Upper Respiratory Infections)) OR (Infections, Upper Respiratory)) OR (Respiratory Infection, Upper)) OR (Nose Diseases[MeSH Terms])) OR (Disease, Nose)) OR (Diseases, Nose)) OR (Nose Disease)) OR (Nasal Diseases)) OR (Disease, Nasal)) OR (Diseases, Nasal)) OR (Nasal Disease)) OR (Nasal Disorders)) OR (Disorder, Nasal)) OR (Disorders, Nasal)) OR (Nasal Disorder)) OR (Pulmonary Disease, Chronic Obstructive[MeSH Terms])) OR (Pulmonary Disease, Chronic Obstructive)) OR (COPD)) OR (Chronic Obstructive Pulmonary Disease)) OR (COAD)) OR (Chronic Obstructive Airway Disease)) OR (Chronic Obstructive Lung Disease)) OR (Airflow Obstruction, Chronic)) OR (Airflow Obstructions, Chronic)) OR (Chronic Airflow Obstructions)) OR (Chronic Airflow Obstruction)) OR (Pleural Diseases[MeSH Terms])) OR (Pleural Diseases)) OR (Disease, Pleural)) OR (Diseases, Pleural)) OR (Pleural Disease)) OR (Respiration Disorders[MeSH Terms])) OR (Respiration Disorders)) OR (Disorder, Respiration)) OR (Disorders, Respiration)) OR (Respiration Disorder)) OR (Respiratory Distress Syndrome, Adult[MeSH Terms])) OR (Respiratory Distress Syndrome, Adult))

	OR (Shock Lung)) OR (Lung, Shock)) OR (ARDS, Human)) OR (ARDSs, Human)) OR (Human ARDS)) OR (Respiratory Distress Syndrome, Acute)) OR (Acute Respiratory Distress Syndrome)) OR (Adult Respiratory Distress Syndrome)) OR (Respiratory Distress Syndrome, Newborn[MeSH Terms])) OR (Respiratory Distress Syndrome, Newborn)) OR (Neonatal Respiratory Distress Syndrome)) OR (Respiratory Distress Syndrome, Infant)) OR (Infantile Respiratory Distress Syndrome)) OR (Pneumonia[MeSH Terms])) OR (Pneumonia)) OR (Pneumonias)) OR (Lobar Pneumonia)) OR (Lobar Pneumonias)) OR (Pneumonias, Lobar)) OR (Pneumonia, Lobar)) OR (Experimental Lung Inflammation)) OR (Experimental Lung Inflammations)) OR (Inflammation, Experimental Lung)) OR (Lung Inflammation, Experimental)) OR (Lung Inflammations, Experimental)) OR (Pneumonitis)) OR (Pneumonitides)) OR (Pulmonary Inflammation)) OR (Inflammation, Pulmonary)) OR (Inflammations, Pulmonary)) OR (Pulmonary Inflammations)) OR (Lung Inflammation)) OR (Inflammation, Lung)) OR (Inflammations, Lung)) OR (Lung Inflammations)) OR (Adenoids[MeSH Terms])) OR (Adenoids)) OR (Adenoid)) OR (Pharyngeal Tonsils)) OR (Tonsil, Pharyngeal)) OR (Pharyngeal Tonsil)) OR (Tonsils, Pharyngeal)) OR (Asthma[MeSH Terms])) OR (Asthma)) OR (Asthmas)) OR (Bronchial Asthma)) OR (Asthma, Bronchial)) OR (Respiratory Distress)) OR (Respiratory infection)) OR (Respiratory tract)) OR (Respiratory infections)) OR (Respiratory diseases)) OR (Pregnancy Complications[MeSH Terms])) OR (Pregnancy Complications)) OR (Complication, Pregnancy)) OR (Pregnancy Complication)) OR (Complications, Pregnancy)
Scopus	#1- (ALL (humans) OR ALL ("Man, Modern") OR ALL ("Modern Man") OR ALL ("Man (Taxonomy)") OR ALL ("Homo sapiens") OR ALL (human) OR ALL ("Dentition permanent") OR ALL ("Permanent Dentition") OR ALL ("Dentition, Secondary") OR ALL ("Secondary Dentition") OR ALL ("Dentition, Adult") OR ALL ("Adult Dentition"))<br>#2- (ALL ("Dental Enamel Hypoplasia") OR ALL ("Hypoplastic Enamel") OR ALL ("Enamel, Hypoplastic") OR ALL ("Enamel Hypoplasia, Dental") OR ALL ("Hypoplasia, Dental Enamel") OR ALL ("Enamel Agenesis") OR ALL ("Ageneses, Enamel") OR ALL ("Agenesis, Enamel") OR ALL ("Enamel Ageneses") OR ALL ("Enamel Hypoplasia") OR ALL ("Enamel Hypoplasias") OR ALL ("Hypoplasia, Enamel") OR ALL ("Hypoplasias, Enamel") OR ALL ("Molar Incisor Hypomineralization") OR ALL (enamel) OR ALL ("Molar Incisor Hypomineralization") OR ALL ("Hypomineralization, Molar Incisor") OR ALL ("Tooth Demineralization") OR ALL ("Tooth Hypomineralization") OR ALL ("Hypomineralization, Tooth") OR ALL ("Hypomineralizations, Tooth") OR ALL ("Tooth Hypomineralizations") OR ALL ("Demineralization, Tooth"))<br>#3- (ALL ("Respiratory Tract Diseases") OR ALL ("Disease, Respiratory Tract") OR ALL ("Diseases, Respiratory Tract") OR ALL ("Respiratory Tract Disease") OR ALL ("Tract Disease, Respiratory") OR ALL ("Tract Diseases, Respiratory") OR ALL (rhinosinusitis) OR ALL ("Acute respiratory infection") OR ALL ("Respiratory disease") OR ALL ("Bronchial Diseases") OR ALL ("Bronchial Disease") OR ALL ("Disease, Bronchial") OR ALL ("Diseases, Bronchial") OR ALL ("Laryngeal Diseases") OR ALL ("Disease, Laryngeal") OR ALL ("Diseases, Laryngeal") OR ALL ("Laryngeal Disease") OR ALL ("Larynx Diseases") OR ALL ("Disease, Larynx") OR ALL ("Diseases, Larynx") OR ALL ("Larynx Disease") OR ALL ("Laryngeal Perichondritis") OR ALL ("Laryngeal Perichondritides") OR ALL ("Perichondritides, Laryngeal") OR ALL ("Perichondritis, Laryngeal") OR ALL ("Lung Diseases") OR ALL ("Disease, Lung") OR ALL ("Diseases, Lung") OR ALL ("Lung Disease") OR ALL ("Pulmonary Disease") OR ALL ("Disease, Pulmonary") OR ALL ("Diseases, Pulmonary") OR ALL ("Pulmonary Diseases") OR ALL ("Respiratory Tract Infections") OR ALL ("Infection,

	Respiratory Tract") OR ALL ("Respiratory Tract Infection") OR ALL ("Respiratory Infections") OR ALL ("Infections, Respiratory") OR ALL ("Infections, Respiratory Tract") OR ALL ("Upper Respiratory Tract Infections") OR ALL ("Infections, Upper Respiratory Tract") OR ALL ("Upper Respiratory Infections") OR ALL ("Infections, Upper Respiratory") OR ALL ("Respiratory Infection, Upper") OR ALL ("Nose Diseases") OR ALL ("Disease, Nose") OR ALL ("Diseases, Nose") OR ALL ("Nose Disease") OR ALL ("Nasal Diseases") OR ALL ("Disease, Nasal") OR ALL ("Diseases, Nasal") OR ALL ("Nasal Disease") OR ALL ("Nasal Disorders") OR ALL ("Disorder, Nasal") OR ALL ("Disorders, Nasal") OR ALL ("Nasal Disorder") OR ALL ("Pulmonary Disease, Chronic Obstructive") OR ALL (copd) OR ALL ("Chronic Obstructive Pulmonary Disease") OR ALL (coad) OR ALL ("Chronic Obstructive Airway Disease") OR ALL ("Chronic Obstructive Lung Disease") OR ALL ("Airflow Obstruction, Chronic") OR ALL ("Airflow Obstructions, Chronic") OR ALL ("Chronic Airflow Obstructions") OR ALL ("Chronic Airflow Obstruction") OR ALL ("Pleural Diseases") OR ALL ("Disease, Pleural") OR ALL ("Diseases, Pleural") OR ALL ("Pleural Disease") OR ALL ("Respiration Disorders") OR ALL ("Disorder, Respiration") OR ALL ("Disorders, Respiration") OR ALL ("Respiration Disorder") OR ALL ("Respiratory Distress Syndrome, Adult") OR ALL ("Shock Lung") OR ALL ("Lung, Shock") OR ALL ("ARDS, Human") OR ALL ("ARDSs, Human") OR ALL ("Human ARDS") OR ALL ("Respiratory Distress Syndrome, Acute") OR ALL ("Acute Respiratory Distress Syndrome") OR ALL ("Adult Respiratory Distress Syndrome") OR ALL ("Respiratory Distress Syndrome, Newborn") OR ALL ("Neonatal Respiratory Distress Syndrome") OR ALL ("Respiratory Distress Syndrome, Infant") OR ALL ("Infantile Respiratory Distress Syndrome") OR ALL (pneumonia) OR ALL (pneumonias) OR ALL ("Lobar Pneumonia") OR ALL ("Lobar Pneumonias") OR ALL ("Pneumonias,Lobar") OR ALL ("Pneumonia,Lobar") OR ALL ("Experimental Lung Inflammation") OR ALL ("Experimental Lung Inflammations") OR ALL ("Inflammation,Experimental Lung") OR ALL ("Lung Inflammation,Experimental") OR ALL ("Lung Inflammations,Experimental") OR ALL (pneumonitis) OR ALL (pneumonitides) OR ALL ("Pulmonary Inflammation") OR ALL ("Inflammation,Pulmonary") OR ALL ("Inflammations,Pulmonary") OR ALL ("Pulmonary Inflammations") OR ALL ("Lung Inflammation") OR ALL ("Inflammation,Lung") OR ALL ("Inflammations,Lung") OR ALL ("Lung Inflammations") OR ALL (adenoids) OR ALL (adenoid) OR ALL ("Pharyngeal Tonsils") OR ALL ("Tonsil,Pharyngeal") OR ALL ("Pharyngeal Tonsil") OR ALL ("Tonsils,Pharyngea") OR ALL (asthma) OR ALL (asthmas) OR ALL ("Bronchial Asthma") OR ALL ("Asthma,Bronchial") OR ALL ("Respiratory Distress") OR ALL ("Respiratory infection") OR ALL ("Respiratory tract") OR ALL ("Respiratory infections") OR ALL ("Respiratory diseases") OR ALL ("Pregnancy Complications") OR ALL ("Complication,Pregnancy") OR ALL ("Pregnancy Complication") OR ALL ("Complications,Pregnancy"))
Web of Science	#1= (humans* OR "Man, Modern" OR "Modern Man" OR "Man (Taxonomy)" OR "Homo sapiens" OR "Dentition permanent" OR "Permanent Dentition" OR "Dentition, Secondary" OR "Secondary Dentition" OR "Dentition, Adult" OR "Adult Dentition")<br>#2- ("Dental Enamel Hypoplasia" OR "Hypoplastic Enamel" OR "Enamel, Hypoplastic" OR"Enamel Hypoplasia, Dental" OR "Hypoplasia, Dental Enamel" OR "Enamel Agenesis" OR "Ageneses, Enamel" OR "Agenesis, Enamel" OR "Enamel Ageneses" OR "Enamel Hypoplasia" OR "Enamel Hypoplasias" OR "Hypoplasia, Enamel" OR "Hypoplasias, Enamel" OR "Molar Incisor Hypomineralization" OR enamel OR "Molar Incisor Hypomineralization" OR "Hypomineralization, Molar Incisor" OR "Tooth

	Demineralization" OR "Tooth Hypomineralization" OR "Hypomineralization, Tooth" OR "Hypomineralizations, Tooth" OR "Tooth Hypomineralizations" OR "Demineralization, Tooth")<br>#3- "Respiratory Tract Diseases" OR "Disease*, Respiratory Tract" OR "Tract Disease*, Respiratory" OR rhinosinusitis OR "Bronchial Disease*" OR "Laryngeal Disease*" OR "Larynx Disease*" OR "Perichondriti*, Laryngeal" OR "Lung Disease*" OR "Pulmonary Disease*" OR "Respiratory Tract Infection*" OR "Respiratory Infection*" OR "Upper Respiratory Tract Infection*" OR "Nose Disease*" OR "Nasal Disease*" OR "Nasal Disorder*" OR "Pulmonary Disease, Chronic Obstructive" OR "Chronic Obstructive Pulmonary Disease" OR "Chronic Obstructive Airway Disease" OR "Chronic Airflow Obstruction*" OR "Pleural Disease*" OR "Respiration Disorder*" OR "Respiratory Distress Syndrome, Adult" OR "Shock Lung" OR "Human ARDS" OR "Acute Respiratory Distress Syndrome" OR "Adult Respiratory Distress Syndrome" OR "Respiratory Distress Syndrome, Newborn" OR "Neonatal Respiratory Distress Syndrome" OR "Respiratory Distress Syndrome, Infant" OR pneumonia* OR "Lobar Pneumonia*" OR "Experimental Lung Inflammation*" OR pneumonitis* OR "Pulmonary Inflammation*" OR "Lung Inflammation*" OR adenoid* OR "Pharyngeal Tonsil*" OR asthma* OR "Bronchial Asthma" OR "Respiratory Distress" OR "Respiratory infection" OR "Respiratory tract" OR "Respiratory infections" OR "Respiratory diseases" OR "Pregnancy Complication*"
The Cochrane Library	#1= (humans) OR ("Man, Modern") OR ("Modern Man") OR ("Man (Taxonomy)") OR ("Homo sapiens") OR (human):ti,ab,kw OR ("Dentition permanent") OR ("Permanent Dentition") OR ("Dentition, Secondary") OR ("Secondary Dentition") OR ("Dentition, Adult") OR ("Adult Dentition")<br>#2- ("Dental Enamel Hypoplasia") OR ("Hypoplastic Enamel") OR ("Enamel, Hypoplastic") OR("Enamel Hypoplasia, Dental") OR ("Hypoplasia, Dental Enamel") OR ("Enamel Agenesis") OR ("Ageneses, Enamel") OR ("Agenesis, Enamel") OR ("Enamel Ageneses") OR ("Enamel Hypoplasia") OR ("Enamel Hypoplasias") OR ("Hypoplasia, Enamel") OR ("Hypoplasias, Enamel") OR ("Molar Incisor Hypomineralization") OR (enamel) OR ("Molar Incisor Hypomineralization") OR ("Hypomineralization, Molar Incisor") OR ("Tooth Demineralization") OR ("Tooth Hypomineralization") OR ("Hypomineralization, Tooth") OR ("Hypomineralizations, Tooth") OR ("Tooth Hypomineralizations") OR ("Demineralization, Tooth")<br>#3- ("Respiratory Tract Diseases") OR ("Disease, Respiratory Tract") OR ("Diseases, Respiratory Tract") OR ("Respiratory Tract Disease") OR ("Tract Disease, Respiratory") OR ("Tract Diseases, Respiratory") OR (rhinosinusitis) OR ("Acute respiratory infection") OR ("Respiratory disease") OR ("Bronchial Diseases") OR ("Bronchial Disease") OR ("Disease, Bronchial") OR ("Diseases, Bronchial") OR ("Laryngeal Diseases") OR ("Disease, Laryngeal") OR ("Diseases, Laryngeal") OR ("Laryngeal Disease") OR ("Larynx Diseases") OR ("Disease, Larynx") OR ("Diseases, Larynx") OR ("Larynx Disease") OR ("Laryngeal Perichondritis") OR ("Laryngeal Perichondritides") OR ("Perichondritides, Laryngeal") OR ("Perichondritis, Laryngeal") OR ("Lung Diseases") OR ("Disease, Lung") OR ("Diseases, Lung") OR ("Lung Disease") OR ("Pulmonary Disease") OR ("Disease, Pulmonary") OR ("Diseases, Pulmonary") OR ("Pulmonary Diseases") OR ("Respiratory Tract Infections") OR ("Infection, Respiratory Tract") OR ("Respiratory Tract Infection") OR ("Respiratory Infections") OR ("Infections, Respiratory") OR ("Infections, Respiratory Tract") OR ("Upper Respiratory Tract Infections") OR ("Infections, Upper Respiratory Tract") OR ("Upper Respiratory Infections") OR ("Infections, Upper Respiratory") OR ("Respiratory Infection, Upper") OR ("Nose Diseases") OR ("Disease, Nose") OR ("Diseases, Nose") OR ("Nose Disease") OR ("Nasal Diseases") OR ("Disease, Nasal") OR ("Diseases, Nasal") OR ("Nasal Disease") OR ("Nasal Disorders") OR ("Disorder, Nasal") OR ("Disorders, Nasal") OR

("Nasal Disorder") OR ("Pulmonary Disease, Chronic Obstructive") OR (copd) OR ("Chronic Obstructive Pulmonary Disease") OR (coad) OR ("Chronic Obstructive Airway Disease") OR ("Chronic Obstructive Lung Disease") OR ("Airflow Obstruction, Chronic") OR ("Airflow Obstructions, Chronic") OR ("Chronic Airflow Obstructions") OR ("Chronic Airflow Obstruction") OR ("Pleural Diseases") OR ("Disease, Pleural") OR ("Diseases, Pleural") OR ("Pleural Disease") OR ("Respiration Disorders") OR ("Disorder, Respiration") OR ("Disorders, Respiration") OR ("Respiration Disorder") OR ("Respiratory Distress Syndrome, Adult") OR ("Shock Lung") OR ("Lung, Shock") OR ("ARDS, Human") OR ("ARDSs, Human") OR ("Human ARDS") OR ("Respiratory Distress Syndrome, Acute") OR ("Acute Respiratory Distress Syndrome") OR ("Adult Respiratory Distress Syndrome") OR ("Respiratory Distress Syndrome, Newborn") OR ("Neonatal Respiratory Distress Syndrome") OR ("Respiratory Distress Syndrome, Infant") OR ("Infantile Respiratory Distress Syndrome") OR (pneumonia) OR (pneumonias) OR ("Lobar Pneumonia") OR ("Lobar Pneumonias") OR ("Pneumonias,Lobar") OR ("Pneumonia,Lobar") OR ("Experimental Lung Inflammation") OR ("Experimental Lung Inflammations") OR (Inflammation,Experimental Lung) OR ("Lung Inflammation,Experimental") OR ("Lung Inflammations,Experimental") OR (pneumonitis) OR (pneumonitides) OR ("Pulmonary Inflammation") OR ("Inflammation,Pulmonary") OR ("Inflammations,Pulmonary") OR ("Pulmonary Inflammations") OR ("Lung Inflammation") OR ("Inflammation,Lung") OR ("Inflammations,Lung") OR ("Lung Inflammations") OR (adenoids) OR (adenoid) OR ("Pharyngeal Tonsils") OR ("Tonsil,Pharyngeal") OR ("Pharyngeal Tonsil") OR ("Tonsils,Pharyngea") OR (asthma) OR (asthmas) OR ("Bronchial Asthma") OR ("Asthma,Bronchial") OR ("Respiratory Distress") OR ("Respiratory infection") OR ("Respiratory tract") OR ("Respiratory infections") OR ("Respiratory diseases") OR ("Pregnancy Complications") OR ("Complication,Pregnancy") OR ("Pregnancy Complication") OR ("Complications,Pregnancy")

LILACS

#1= (humans) OR ("Man, Modern") OR ("Modern Man") OR ("Man (Taxonomy)") OR ("Homo sapiens") OR (human):ti,ab,kw OR ("Dentition permanent") OR ("Permanent Dentition") OR ("Dentition, Secondary") OR ("Secondary Dentition") OR ("Dentition, Adult") OR ("Adult Dentition")

#2- ("Dental Enamel Hypoplasia") OR ("Hypoplastic Enamel") OR ("Enamel, Hypoplastic") OR("Enamel Hypoplasia, Dental") OR ("Hypoplasia, Dental Enamel") OR ("Enamel Agenesis") OR ("Ageneses, Enamel") OR ("Agenesis, Enamel") OR ("Enamel Ageneses") OR ("Enamel Hypoplasia") OR ("Enamel Hypoplasias") OR ("Hypoplasia, Enamel") OR ("Hypoplasias, Enamel") OR ("Molar Incisor Hypomineralization") OR (enamel) OR ("Molar Incisor Hypomineralization") OR ("Hypomineralization, Molar Incisor") OR ("Tooth Demineralization") OR ("Tooth Hypomineralization") OR ("Hypomineralization, Tooth") OR ("Hypomineralizations, Tooth") OR ("Tooth Hypomineralizations") OR ("Demineralization, Tooth")

#3- ("Respiratory Tract Diseases") OR ("Disease, Respiratory Tract") OR ("Diseases, Respiratory Tract") OR ("Respiratory Tract Disease") OR ("Tract Disease, Respiratory") OR ("Tract Diseases, Respiratory") OR (rhinosinusitis) OR ("Acute respiratory infection") OR ("Respiratory disease") OR ("Bronchial Diseases") OR ("Bronchial Disease") OR ("Disease, Bronchial") OR ("Diseases, Bronchial") OR ("Laryngeal Diseases") OR ("Disease, Laryngeal") OR ("Diseases, Laryngeal") OR ("Laryngeal Disease") OR ("Larynx Diseases") OR ("Disease, Larynx") OR ("Diseases, Larynx") OR ("Larynx Disease") OR ("Laryngeal Perichondritis") OR ("Laryngeal Perichondritides") OR ("Perichondritides, Laryngeal") OR ("Perichondritis,

	Laryngeal”) OR ("Lung Diseases”) OR ("Disease, Lung”) OR ("Diseases, Lung”) OR ("Lung Disease”) OR ("Pulmonary Disease”) OR ("Disease, Pulmonary”) OR ("Diseases, Pulmonary”) OR ("Pulmonary Diseases”) OR ("Respiratory Tract Infections”) OR ("Infection, Respiratory Tract”) OR ("Respiratory Tract Infection”) OR ("Respiratory Infections”) OR ("Infections, Respiratory”) OR ("Infections, Respiratory Tract”) OR ("Upper Respiratory Tract Infections”) OR ("Infections, Upper Respiratory Tract”) OR ("Upper Respiratory Infections”) OR ("Infections, Upper Respiratory”) OR ("Respiratory Infection, Upper") OR ("Nose Diseases”) OR ("Disease, Nose”) OR ("Diseases, Nose”) OR ("Nose Disease”) OR ("Nasal Diseases”) OR ("Disease, Nasal”) OR ("Diseases, Nasal”) OR ("Nasal Disease”) OR ("Nasal Disorders”) OR ("Disorder, Nasal”) OR ("Disorders, Nasal”) OR ("Nasal Disorder”) OR ("Pulmonary Disease, Chronic Obstructive”) OR (copd) OR ("Chronic Obstructive Pulmonary Disease”) OR (coad) OR ("Chronic Obstructive Airway Disease”) OR ("Chronic Obstructive Lung Disease”) OR ("Airflow Obstruction, Chronic”) OR ("Airflow Obstructions, Chronic”) OR ("Chronic Airflow Obstructions”) OR ("Chronic Airflow Obstruction”) OR ("Pleural Diseases”) OR ("Disease, Pleural”) OR ("Diseases, Pleural”) OR ("Pleural Disease”) OR ("Respiration Disorders”) OR ("Disorder, Respiration”) OR ("Disorders, Respiration”) OR ("Respiration Disorder”) OR ("Respiratory Distress Syndrome, Adult”) OR ("Shock Lung”) OR ("Lung, Shock”) OR ("ARDS, Human”) OR ("ARDSs, Human”) OR ("Human ARDS”) OR ("Respiratory Distress Syndrome, Acute”) OR ("Acute Respiratory Distress Syndrome”) OR ("Adult Respiratory Distress Syndrome”) OR ("Respiratory Distress Syndrome, Newborn”) OR ("Neonatal Respiratory Distress Syndrome”) OR ("Respiratory Distress Syndrome, Infant”) OR ("Infantile Respiratory Distress Syndrome") OR (pneumonia) OR (pneumonias) OR ("Lobar Pneumonia”) OR ("Lobar Pneumonias”) OR ("Pneumonias,Lobar”) OR ("Pneumonia,Lobar”) OR ("Experimental Lung Inflammation”) OR ("Experimental Lung Inflammations”) OR (Inflammation,Experimental Lung) OR ("Lung Inflammation,Experimental”) OR ("Lung Inflammations,Experimental”) OR (pneumonitis) OR (pneumonitides) OR ("Pulmonary Inflammation”) OR ("Inflammation,Pulmonary”) OR ("Inflammations,Pulmonary”) OR ("Pulmonary Inflammations”) OR ("Lung Inflammation”) OR ("Inflammation,Lung”) OR ("Inflammations,Lung”) OR ("Lung Inflammations”) OR (adenoids) OR (adenoid) OR ("Pharyngeal Tonsils”) OR ("Tonsil,Pharyngeal”) OR ("Pharyngeal Tonsil”) OR ("Tonsils,Pharyngea”) OR (asthma) OR (asthmas) OR ("Bronchial Asthma”) OR ("Asthma,Bronchial”) OR ("Respiratory Distress”) OR ("Respiratory infection”) OR ("Respiratory tract”) OR ("Respiratory infections”) OR ("Respiratory diseases”) OR ("Pregnancy Complications”) OR ("Complication,Pregnancy”) OR ("Pregnancy Complication”) OR ("Complications,Pregnancy")
OpenGrey	Humans AND “Molar Incisor Hypomineralization” AND “Respiratory disease”
Google Scholar	Humans+“Molar Incisor Hypomineralization”+“Respiratory disease”-review
